# A stream classification system for the conterminous United States

**DOI:** 10.1038/sdata.2019.17

**Published:** 2019-02-12

**Authors:** Ryan A. McManamay, Christopher R. DeRolph

**Affiliations:** 1 Environmental Sciences Division, Oak Ridge National Laboratory, Oak Ridge, TN 37831, USA

**Keywords:** Freshwater ecology, Environmental chemistry

## Abstract

Stream classifications are important for understanding stream ecosystem diversity while also serving as tools for aquatic conservation and management. With current rates of land and riverscape modification within the United States (US), a comprehensive inventory and evaluation of naturally occurring stream habitats is needed, as this provides a physical template upon which stream biodiversity is organized and maintained. To adequately represent the heterogeneity of stream ecosystems, such a classification needs to be spatially extensive where multiple stream habitat components are represented at the highest resolution possible. Herein, we present a multi-layered empirically-driven stream classification system for the conterminous US, constructed from over 2.6 million stream reaches within the NHDPlus V2 stream network. The classification is based on emergent natural variation in six habitat layers meaningful at the stream-reach resolution: size, gradient, hydrology, temperature, network bifurcation, and valley confinement. To support flexibility of use, we provide multiple alternative approaches to developing classes and report uncertainty in classes assigned to stream reaches. The stream classification and underlying data provide valuable resources for stream conservation and research.

## Background & Summary

Classification systems reveal the structure and relationships among groups of objects, and in doing so, they help us understand complex systems by drawing inferences about the laws that govern those relationships^1^. For instance, stream classifications are often based on commonalities in hydrologic variation^2^, thermal regimes^3^, or geomorphic properties^4,5^. As such, stream classifications are fundamentally important in understanding the diversity of stream ecosystems across large regions^6^ and their role in structuring biological communities^7^. However, stream classifications are also practically important to management, such as serving as conservation planning units^8^, prioritizing conservation and restoration^9^, stratifying environmental monitoring programs^10^, providing predictive variables for species distribution modeling^11^, and identifying reference sites to inform monitoring^12^.

While the approach to developing a stream classification rests upon its intended objectives for use^30^, there are several design principles of classifications that we believe maximize the application breadth for stream research and management^6^. These include developing classifications that are: 1) at the stream-reach resolution, 2) based on multiple layers of habitat components, 3) spatially contiguous and comprehensive, 4) inductive (i.e. emergent properties), 5) physically-based, and 6) representative of the least disturbed condition^6^. We describe each of these principles briefly.

First, stream habitats are shaped by two predominant forces: the physio-climatic properties of the landscapes they drain^13,14^ and the longitudinal and lateral advection of materials^15,16^. Accordingly, stream-reaches are an ideal spatial resolution that captures both local and upstream processes^17–19^ and are best equipped to understand the regional-to-local heterogeneity of riverscapes^7^. Second, to help understand and communicate the multivariate nature of lotic systems, streams have been conceptualized as a series of building blocks representing different components of the ecosystem (e.g., hydrology, geomorphology)^20^. Multi-layered approaches to classification preserve the identify of these building blocks, each of which have different roles in structuring ecological communities or understanding stream responses to natural or human disturbances^6,20^. Third, classifying all observations ensures classifications are comprehensive of all potential types and not biased by the availability of information^21^; however, this induces a tradeoff between developing classifications based on direct measures of stream behavior versus environmental regionalization (i.e. deductive), as direct observations often have limited spatial coverage^22,23^. Hence, the fourth principle: Inductive approaches that rely on direct empirical observations (e.g., discharge) more accurately represent emergent patterns of stream behavior than deductive approaches that use regionalization or indirect environmental surrogates to represent variation in streams^22^. Although there are a few ways to reconcile these divergent endpoints (e.g., novel deductive regionalization-hybrid classification approaches^23^), a straightforward approach is to use predictive models to extrapolate direct measures of stream behavior to all stream reaches. Fifth, physically-based classifications, as opposed to biologically informed classifications, preserves mechanistic linkages between physical process, stream responses to disturbance, and the structure of ecological dynamics^6^. Rendering class partitions based on biological discriminatory power shifts the scale-relevance of subsequent classifications towards the availability of biological data and selected taxonomic groups, which could minimize application breadth. Finally, classes developed based on the reference or least disturbance condition are amenable to guiding restoration and management^12^.

The above principles are a stark contrast to the many previous national-scale stream classification efforts, which have either classified discrete observations (e.g., stream monitoring points)^2^, used deductive approaches for grouping streams^10,24^, and/or classified singular, as opposed to multiple, habitat components, primarily hydrology^22^. While these approaches have enriched our understanding of stream function, they are limited in their ability to comprehensively represent the emergent properties of stream ecosystems and their habitat components across large regions^6,7^. Herein, we describe an inductive, multi-layered stream classification system dataset for stream reaches within the conterminous United States where we followed the six design principles. The Stream Classification System (SCS) is constructed from the NHDPlus V2 stream reach network (http://www.horizon-systems.com/NHDPlus/index.php), a spatial framework of over 2.6 million stream reaches within the conterminous US (CONUS). This effort builds off previous efforts to construct an analogous stream classification product for the Eastern United States^6^. To our knowledge, a comparable stream classification of this scope and resolution has not been documented in the literature, but provides a valuable resource for stream management, conservation, and research applications.

## Methods

### Overview of approach

Within the SCS, stream habitat building blocks are represented as a series of layers, each of which represent different categories of physical characteristics (e.g., size, gradient). Each layer is comprised by multiple classes (e.g., headwater, creek, low gradient, high gradient). Layers were constructed using inductive approaches based on patterns in empirical data, as opposed to deductive approaches reliant upon landscape regionalization. Sources of empirical data used to derive stream classes are provided in Table 1. Through previous reviews and solicitation from a body of conservationists and stream ecologists^6,25^, we selected six stream habitat layers that could be mapped at the stream reach resolution and were hypothesized to exert strong controls on ecological function and ecological community composition. These included (in order of decreasing ecological importance): size, gradient, hydrology, temperature, stream network bifurcation, and valley confinement.

A major consideration in selecting layers and determining partitions among classes was the availability of documented methods for classification approaches and thresholds among classes. Hence, we preferentially selected layers supported by pre-existing and published classifications or if previous classifications were unavailable, we relied on literature to determine breaks and thresholds to partition values (e.g., gradient) into classes when available. Because classification outcomes are influenced by the approach taken, we used multiple alternative approaches, if available, in developing classes within layers.

### Predictor Variable Compilation

Information on size, gradient, and network bifurcation were derived from the NHDPlus V2 dataset. However, discrete *in situ* observations of hydrology, temperature, and river channel characteristics (valley confinement) required that we develop models to extrapolate these classes to the stream reach level. A total of 66 landscape, climate, topographic, and soil variables were assembled for drainage basins contributing to each stream gaging station and for the entire drainage network upstream of every stream reach in the US (Table 2 (available online only)). Of these, 44 were provided by Stream Cat database^26^ (https://www.epa.gov/national-aquatic-resource-surveys/streamcat), 21 from the NHDPlus V2 dataset, and one from WorldClim (http://worldclim.org/version2) (Table 2 (available online only)). In approximately 2% of observations, values were missing for variables summarized for drainage networks above each stream reach (primarily StreamCat data). We used the Multivariate Imputation by Chained Equation (MICE) package in the R programming environment^27^ to estimate the most probable values for missing variables based on values present for other variables. For each variable with missing values, we specified a binary matrix indicating which subset of predictors should be used to estimate missing values during imputation. Separate Predictive Mean Matching models were developed for each incomplete variable^27^.

### Size

In comparison to other classes, developing classification schemes for size and gradient did not rely on *in situ* observations or predictive model development (e.g., hydrology). We used two size-relevant variables available through the NHDPlus V2 dataset to provide alternative classifications of stream size: Strahler stream order and mean annual discharge (representative of conditions of minimal human impact). Stream order depicts the dendritic nature of stream environments^28^ and is commonly used to characterize the frequency distribution of stream sizes over large regions or globally^29^. Limitations of stream order, however, are that order can be influenced by the scale of mapped hydrography^30^ and discharge may vary widely across climatic regimes for a given order. Likewise, using drainage area to characterize size can also be problematic, as discharge per unit area will also range dramatically across regions of widely varying climate^30^. Alternatively, a stream’s size can be characterized by the flow it carries. However, this requires determining a standardized approach to partition classes based on discharge. Because geometric laws governing stream organization (e.g., frequency, stream length, drainage area) are based upon stream order^31^, order provides a universal physical template to partition continental wide variation in discharge based on consistent thresholds. To develop a discharge-based size classification, we calculated the median discharge for all NHDPlus V2 stream reaches according to Strahler stream order and then used mid-points between these values to create discharge breaks as size class thresholds. (Note: variables used in the hydrologic classification are standardized by mean annual discharge and thus, are not influenced by river size).

### Gradient

Gradient values (i.e., stream bed slope) were also provided as an attribute of NHDPlus V2 flowlines. Stream slopes were measured for each flowline as the proportion of rise in elevation over streamline distance^32^. Smoothed elevation data were derived from 10-m digital elevation models (DEMs) for the nation. Maximum and minimum elevations were used to determine rise, which was divided by the total length of the flowline. To our knowledge, the most widely-used gradient thresholds are provided Rosgen^4^, who distinguishes channel morphologies based on gradient, width-to-depth ratios, entrenchment, and sinuosity. Multiple stream classification efforts have also relied on these gradient thresholds to partition classes as well^6,9,25^. We adopted these breaks to develop gradient types and mapped those to stream reaches.

### Hydrology

Over the past two decades, numerous hydrologic classifications at regional to global scales have been developed from discrete observations of streamflow monitoring stations^2,18,33^. In general, developing inductive hydrologic classifications requires assembling *in situ* observations of discharge, summarizing discharge into hydrologic statistics, and then clustering observations based on similarities in hydrologic properties^22^. Recently, McManamay *et al.*^34^ developed a hydrologic classification for the entire US based on natural streamflow patterns at 2,600 US Geological Survey (USGS) stream gaging stations with upstream watersheds representing the least disturbed condition for their respective region. Following decomposition of 110 hydrologic statistics into 13 component scores using Principal Components Analysis (PCA), stream gages were probabilistically assigned to 1 of 15 hydrologic classes using optimal Gaussian mixed model clustering algorithms determined using Bayesian inference^34^. These classes represent variation in hydrologic patterns as opposed to variation in discharge volume, as all magnitude-related hydrologic statistics were standardized by mean daily flow prior to PCA and clustering.

This fuzzy-style of classification (i.e., soft clustering) is flexible in that it characterizes streams as theoretically sharing membership among many clusters^33,35^. In contrast, “hard” clustering techniques, such as distance-based hierarchical agglomerative methods (e.g. Ward’s method)^36^, are relatively straightforward, easier to understand, and produce nested and crisp memberships^22^. Thus, we used Ward’s agglomerative method to cluster the 2600 USGS gages using the 13 PC scores and then determined a series of optimal numbers of clusters based on visual examination of the dendrogram.

All USGS stream gages were spatially joined to NHDPlus V2 stream reaches. Using predictor variables in Table 2 (available online only), we constructed random forest classification models^37^ in the R programming environment to predict hydrologic class membership and then extrapolated hydrologic classes to all NHDPlus V2 stream reaches.

### Temperature

Compared to hydrology, temperature classifications are less common^3,38,39^, possibly due to scarcer temperature data compared to discharge. Recently, Maheu *et al.*^3^ grouped approximately 130 gaging stations (representative of reference conditions) across the US into different types of thermal regimes based on a several statistics describing magnitude and variation. This multivariate approach provides a multivariate alternative to the univariate summer temperature classes that we generated. Locations of gages used in the Maheu *et al.* classification were acquired from the authors and were spatially joined to NHDPlus V2 stream reaches. Using 65 of the predictor variables, we developed a random forest model to Maheu *et al.* classes to stream reaches across the US. Because temperature is a function of river size, we excluded Qwsa from the model (i.e. mean annual flow divided by drainage area).

As an alternative, we developed a simple temperature classification based on naturally occurring average summer water temperature values. Multiple studies suggest that divergent thermal regimes in streams are primarily influenced by natural variation in summer temperature (July–August averages) values^3,40,41^. Additionally, summer-time temperature values are among the most readily available data from public and non-public sources. We compiled stream water temperature data for 5,907 sites from multiple sources, including Deweber & Wagner^41^ (n = 2893), Hill *et al.*^40^ (n = 566), USGS gauges with daily records (n = 2184), USGS seasonal field monitoring (n = 240), and other temperature data from loggers deployed by agencies (n = 24) (Table 1). Determining adequate record length for temperature data required striking a balance between minimizing uncertainty in July–August averages with having too few samples for adequate regional representation. For instance, Jones and Schmidt^42^ provided recommendations for record lengths required to adequately minimize uncertainty in estimating thermal regime metrics; however, following this guidance would have reduced the above USGS records alone (n = 2424) by 70 to 90%. Furthermore, Jones and Schmidt’s assessment included monthly maxima, minima, and range metrics, whereas our analysis relied on a coarser bi-monthly average metric (July–August), which we deem less susceptible to year-to-year variation than temperature extremes (Supplementary File 1). Using 22 USGS gages across the US and confidence bands from Jones and Schmidt, we estimate that 1–2 seasons of data could reliably estimate mean July–August temperatures within 1 °C at 80% and 90% confidence, respectively (Supplementary File 1). We screened sites to ensure the period of record fell within 1995 to 2015 and data was available for at least 60 consecutive days in July and August.

All temperature sites were spatially joined to NHDPlus V2 stream reaches. We then determined reference conditions for monitoring sites using indicators of land disturbance and upstream dam regulation. Land disturbance was evaluated using the National Fish Habitat Partnership (NFHP) 2015 habitat assessment, which provides habitat degradation scores ranging from “very low” to “very high” disturbances within NHDPlus stream reach segments^43^. We evaluated the degree of upstream regulation by impoundments using the degree of regulation (DOR) (% of annual discharge stored by upstream dams)^44^, provided by StreamCat. Temperature monitoring stations with risk assessment scores as “very low” or “low” and DOR < 4% (indicating little influence of reservoirs^44,45^) were determined representative of reference conditions, which resulted in 1764 sites that also met our record length criteria. Of these, 70% of observations were obtained from Deweber & Wagner^41^ (n = 1211) or Hill *et al.*^40^ (n = 33). Of the remaining 520 observations, 71.7% had at least 2 seasons of data.

Using the same predictor ensemble above, we developed random forests to predict summer temperatures for reference sites and then extrapolated those values to all NHD stream reaches. We used breaks in the frequency distribution of US water temperatures to partition summer temperatures into classes. Using estimated summer-time temperature values for all stream reaches, we used a Jenks Natural Breaks^46^ procedure to partition temperatures into 2 to 20 classes and then relied upon optimal goodness-of-fit and tabular accuracy to determine the most parsimonious number of classes explaining the majority of information. In the absence of a justified approach for physically-based partitioning of classes, the Jenks method is optimal for univariate clustering of spatial information as it seeks to minimize variation within classes while maximizing variance among classes^46^.

### Network Bifurcation

Whereas stream size captures the longitudinal variation of ecological functions along a stream’s continuum^15^, tributary junctions and stream divergences are also important as they create discontinuities in longitudinal processes^47^. Stream junctions, specifically the differential sizes of streams that comprise junctions, have large influences on habitat and biological diversity^48^. Additionally, ecological community composition can dramatically change with proximity to stream junctions^49^. To capture differences in network configurations and situations of divergence, we created two bifurcation classes. First, we created classes that accounted for different size combinations of tributaries forming a confluence at the upstream end of each stream reach. Second, we developed classes indicating stream reaches as main or secondary channels below divergences and where streams received flow from upstream divergences.

**Figure 1:**
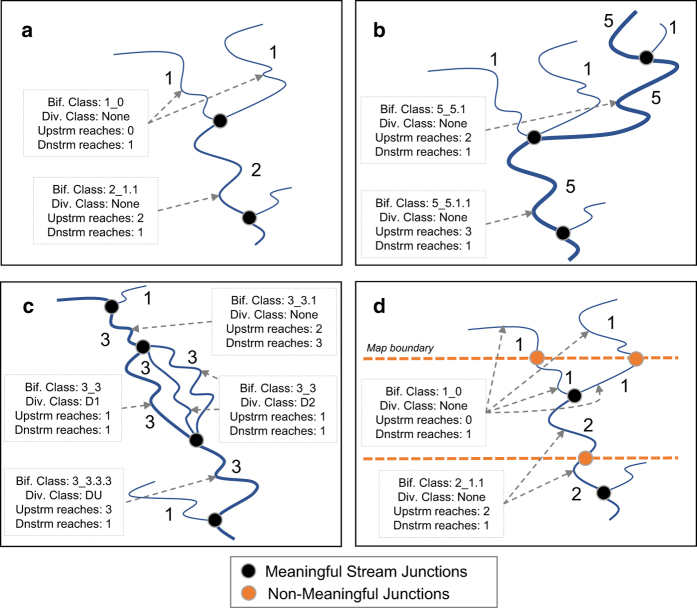
Conceptual diagram of various scenarios of stream network bifurcation and divergence. For each scenario, reaches are labeled according to their Strahler stream order. Bifurcation (Bif.) classes, divergence (Div.) classes, and the number of upstream and downstream reaches are noted. Naturally-occurring (i.e. meaningful) stream junctions are distinguished from non-meaningful stream reach junctions arising from quadrangle map boundaries. Scenarios include (**a**) a common, simplified stream junction, (**b**) a more complex junction with more than 2 upstream contributing reaches, (**c**) a situation of stream divergence, and (**d**) non-meaningful stream junctions arising from map boundaries. In the case of (**d**), reaches immediately occurring downstream of non-meaningful junctions are assigned to the same class as their upstream neighboring reach.

Most individual stream reaches within the NHDplus V2 dataset represent distinct hydrologic features of river networks defined by stream origins, tributary confluences, and intersections with lakes and reservoirs^50^. Topological relationships among NHDplus V2 stream reaches are provided in a “from-to” table defining the upstream reaches contributing to a given reach (i.e., from) and the downstream reach receiving flow (i.e., to). Using the “from-to” table, the combinations of different Strahler stream orders at the upstream end of each reach were combined to create a tributary-mainstem combination. For instance, the confluence of a 1^st^ order and 2^nd^ order tributaries at the upstream end of a 2^nd^ order system would yield the following class: 2.12 (Fig. 1a). In the majority of cases, only 2 tributaries occurred upstream. However, in rare cases or situations of divergence, 3 or more tributaries merge upstream above a reach and we included up to four upstream orders (e.g., Fig. 1b, 5.511). In some cases, stream reaches receive flow from multiple upstream channel divergences, i.e. splits of one reach into two or more channels in the downstream direction (Fig. 1c). Because these channels are assigned a stream order and create junctions that mimic tributary confluences, classifying network bifurcation requires including channel divergences as a type of confluence. In cases of channel divergence, NHDplus V2 designates reaches as main (D1) or secondary (D2) channels (Fig. 1c). We used the from-to table to identify stream reaches that were immediately below confluences of channel divergences (DU), as to distinguish these from tributary confluences. After accounting for these divergences, we observed situations of non-sensical tributary junctions (e.g., 5_5.5) that arose because NHDplus V2 did not appropriately designate all situations of channel divergence. Because it was difficult to determine whether each of these reaches were divergent channels or reaches receiving flow from divergent channels, we assigned these reaches to a generic divergence class (D).

Although most tributary junctions in NHDPlus V2 are hydrologically relevant, a subset of reach junctions were split at unmeaningful points, such as quadrangle map boundaries, during digitization^50^ (Fig. 1d). In the case of bifurcation classes and divergences, these splits would lead to non-sensical junctions. To correct these instances, Wieferich *et al.*^51^ produced an Ecological Reach Identification Table that assigned split reaches to common ecological identifiers. In these cases, we assigned all reaches belonging to the same ecological unit with the bifurcation and divergence class of the upstream-most reach (Fig. 1d).

### Valley Confinement

The degree to which valleys control the lateral migration of river channels is indicative of the strength of interaction between rivers and their floodplain. We delineated unconstrained valley bottoms (i.e., polygons) for all NHDPlus V2 stream reaches using the Valley Confinement Algorithm (VCA) tool^52^ in ArcMap 10.3. VCA estimates bankfull depth of the stream channel using an empirical function based on regional precipitation data (http://www.prism.oregonstate.edu/normals) and drainage area for each stream reach^53^. Nagle *et al.*^52^ suggested 5X bankfull depth to determine flood height, which we also deemed appropriate given the spatial resolution of NHDplus and 30-m DEM data (https://nationalmap.gov/elevation.html) for surrounding topography. Based on the surrounding terrain characterized via DEMs, the VCA program used an algorithm to intersect flood height with the surrounding hillslope. Waterbodies were used to avoid delineation of valley bottoms in inundated areas.

Once valley bottoms were delineated, thresholds are required to classify stream reaches as unconfined, confined, or an intermediate level. For example, a valley bottom may not encompass an entire stream reach or may not extend laterally a sufficient distance beyond stream banks to be classified as unconfined. This requires an estimate of river width for each stream reach. We compiled both *in situ* field and remote sensing observations from >52,000 sites to develop an empirical model to predict river width for all stream reaches in the CONUS. Field observations of river width were derived from Environmental Protection Agency’s National Rivers and Streams Assessment (n = 852) (https://www.epa.gov/national-aquatic-resource-surveys/nrsa), a literature review of stream widths (n = 243)^29^, and the North American River Width Data Set (n = 50,230) (http://gaia.geosci.unc.edu/NARWidth/). However, these datasets largely missed small headwater streams and intermittent systems. To ensure we properly estimated width for these stream types, stream reaches were stratified by size (see Size classification) and a random subset (n = 407) were selected from the entire US stream reach population. Aerial imagery was used to estimate river width at the midpoint, upstream, and downstream ends of each reach, and then calculate an average width. Random forest models were used to predict river width and extrapolate estimates to all stream reaches. River width estimates were then used to generate polygon buffers around all streamlines.

We overlaid river widths and valley bottoms to determine valley constraint status. Hall *et al.*^53^ considered stream reaches unconfined if the width of the floodplain valley is at least four times the width, whereas stream channels with moderate floodplain interaction have floodplain-to-bankfull width ratios >2^4^. Beyond the lateral extent of floodplains, our assessment of confinement also required examining the length of each stream reach covered by valley bottoms. Stream reaches were classified as “unconfined” if a valley bottom covered at least 50% of the stream reach length and had a width at least four times that of the river width. “Moderately confined” stream reaches had valley bottoms with widths >4X river width but only covered 25–50% of the stream reach length, or if greater than 50% coverage of stream length, valley bottoms had floodplain:river width ratios between 2 and 4. All other stream reaches were defined as “confined.”

## Data Records

The US SCS is available to the public by a downloadable link on the Oak Ridge National Laboratory National Hydropower Asset Assessment Program (https://nhaap.ornl.gov/us-sct) and through figshare (Data Citation 1). A list of datasets and their variables are provided in Table 3. Variables include the categorical values resulting from the classification, continuous or nominal variables used in developing the classes, or measures of probability of class membership (Table 3). Data for each dataset category (e.g., Size and Gradient) are provided as a series of .csv files, each pertaining to one of four regions of the US split by major basins (East, Upper Mississippi, Lower Mississippi, and West). All datasets include the Common Identifier (COMID) to uniquely identify stream reaches and to cross-reference the NHDPlus V2 dataset.

## Technical Validation

Validation of the stream classification layers was assessed using at least two or more of the following approaches depending on the layer: 1) class partitioning results and associated diagnostics (all layers), 2) error and variation explained in models used to derive values underlying classes (temperature, confinement, i.e. river width), 3) misclassification rates of models used to predict class membership in stream reaches (hydrology, temperature), 4) relative importance of variables used in models (hydrology, temperature, river width), and 5) sample size distribution of stream reaches among classes (all layers). Sample sizes (number of reaches) and cumulative stream length according to different classes are provided in Supplementary File 2. The NHDPlus V2 dataset consists of 2.69 million stream reaches, which constitute 5.195 million km of stream length. Assigning class to all stream reaches was not possible because geospatial variables are missing for some reaches, despite our attempt to impute missing values. This arises because streams are braided or consist of artificial channels, which prevents network routing to accumulate geospatial information. The number of reaches lacking class assignment varied according to layer and depended on which variables were required for deterministically partitioning classes or which variables were incorporated into final random forest models. Sample sizes lacking class assignment varied from 12,800 reaches (confinement) to 98,000 reaches (hydrologic classes) were unavailable for classification due to missing predictor variables. Unclassified reaches constituted <1.2% of total stream length in the US.

**Figure 2:**
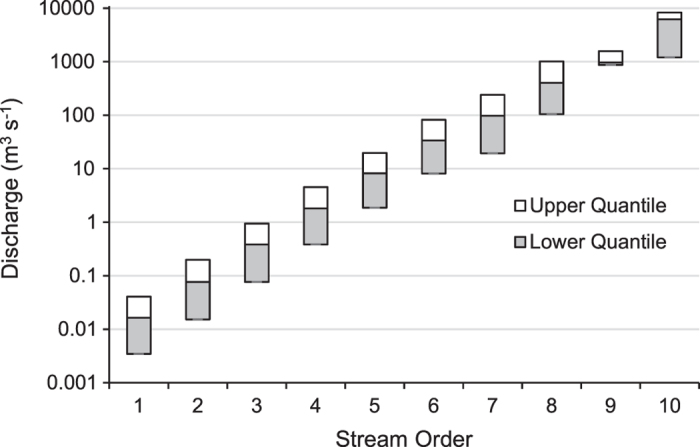
Thresholds for determining partitions between size classes. Box plots (upper and lower quantiles) of discharge according to stream order. Class breaks represent average values between corresponding medians for each stream order.

**Figure 3:**
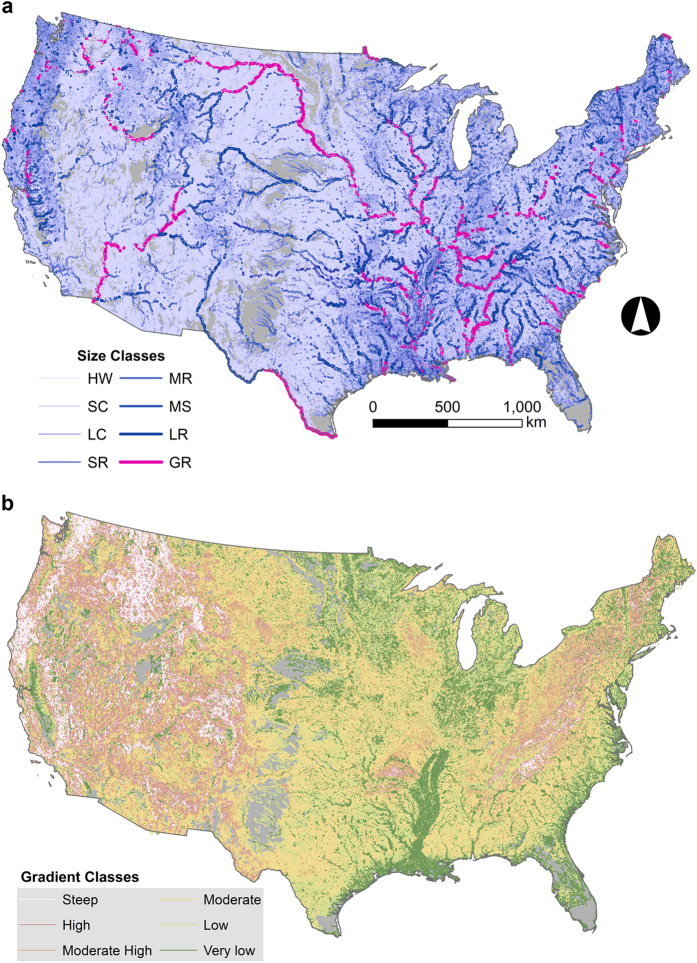
Size and gradient stream classes of the conterminous US. (**a**) Eight size classes based on discharge values mapped to stream reaches. (**b**) Size gradient values mapped to stream reaches.

Values of stream order, discharge, and stream reach slope used to characterize size and gradient layers were obtained from NHDPlus V2 datasets, and thereby incorporate any error and uncertainty arising from remote sensing data used to derive those values^32^. Median and interquartile ranges of discharge values ranged widely among stream orders, which substantiated the limitations of using stream order as a universal measure of river size (Fig. 2). Midpoints between median values of discharge minimized overlap in discharge values among classes (Fig. 2). Class partition thresholds are provided in Table 4. As documented previously^29^, the frequency of stream reaches among size classes and stream orders displayed an exponential decay distribution where the majority of reaches were classified as headwater (1^st^ order systems) and the largest systems were the most infrequent (Fig. 3a, Supplementary File 2). The majority streams had moderate-high gradients (34% of stream length), followed by low gradient (23%), and very low gradient (15%) types (Fig. 3b, Supplementary File 2).

**Figure 4:**
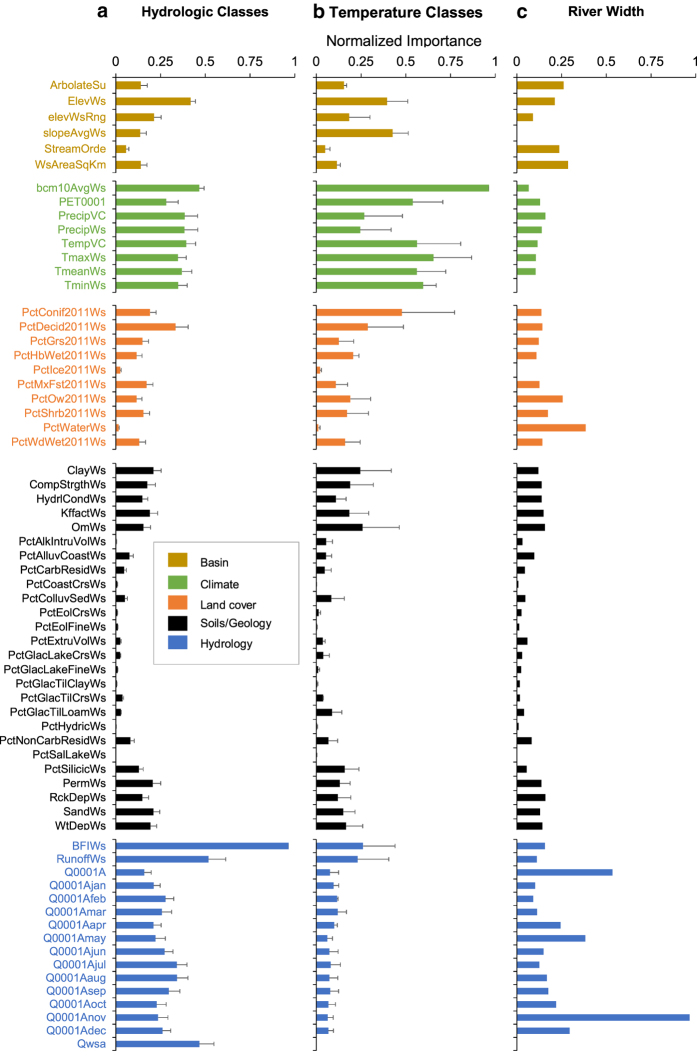
Importance of different predictors used in random forest models. Random forests were used to predict (**a**) hydrologic classes, (**b**) temperature classes or average July—August temperature, or (**c**) river width. Normalized importance refers to node impurity values for the Gini index (classification) or mean-squared error (regression) that are scaled from 0 to 1 using (max – x_i_)/(max (x) – min(x)). Normalized importance was averaged across all random forest models for hydrologic classes and temperature. Error bars represent 1 SE. Note: only 1 random forest model was developed for river width.

**Figure 5:**
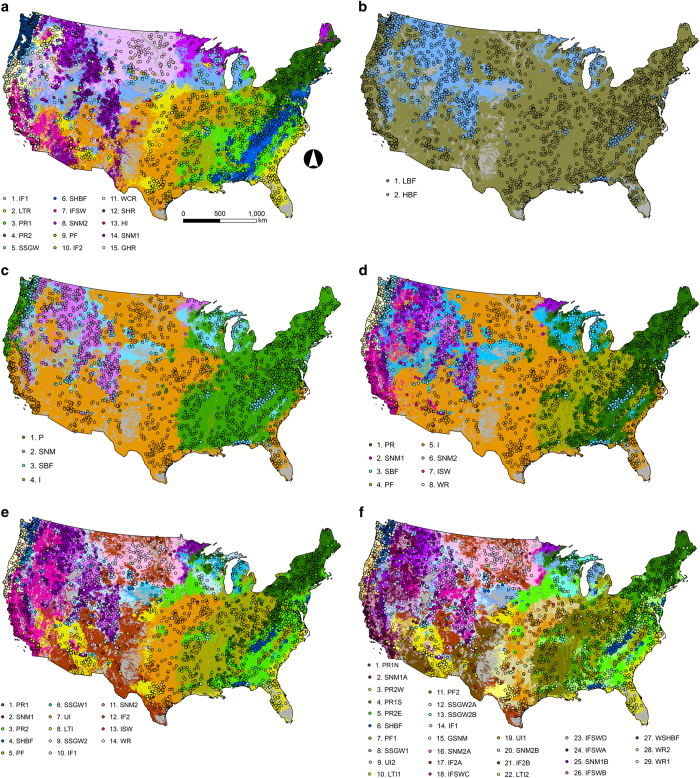
Maps of hydrologic classes assigned to stream reaches in the conterminous US. Different clustering approaches used developed hydrologic classes at stream gauges (points) were mapped to stream reaches including: (**a**) Fifteen gaussian mixture model classes, and several Ward’s agglomerative clustering solutions for (**b**) two, (**c**) four, (**d**) eight, (**e**) fourteen, and (**f**) thirty classes. Acronyms for classes are described in Tables 5 and 6.

Hydrologic classes produced via Gaussian mixture modeling were previously available from McManamay *et al.*^34^ (Table 5), whereas the Ward’s agglomerative procedure required determining numbers of hydrologic classes. Based on visual inspection of dendrograms and reductions in sum-of-squared variation within clusters, we selected cluster solutions representing 2, 4, 8, 14, and 29 different hydrologic classes (Supplementary File 3). The nested hierarchy of these resultant classes are provided in Table 6 and dendrograms are provided in Supplementary File 3. Random forest models predicting class membership resulted in out-of-bag (OOB, i.e. cross validation sample) misclassification rates ranging from 5 to 34% (or 66%–95% accuracies), depending on the classification (Table 7). In general, variables with the highest normalized importance in random forests used to predict hydrologic classes were hydrologic variables and climate variables (Fig. 4); however, selected basin characteristics (e.g., elevation), land cover (deciduous forest), and soil/geology variables (permeability) were also important (Fig. 4). Median probabilities (i.e., proportion of majority votes) of the predominant class membership assigned to individual reaches ranged from 0.34 to 0.91, depending on the cluster approach (Table 7). While seemingly low, these probabilities were considerably higher than expected probabilities for each solution (Table 7). In general, 80% of streams were classified as “low” baseflow systems compared to high baseflow systems (Fig. 5a–f, Supplementary File 2). Additionally, almost 50% of streams had some degree of intermittency (Fig. 5a–f, Supplementary File 2). The most predominant hydrologic types were streams with flashy or intermittent hydrology and lower baseflows, followed by perennial runoff types and then stable baseflow types (Fig. 5a–f, Supplementary File 2).

**Figure 6:**
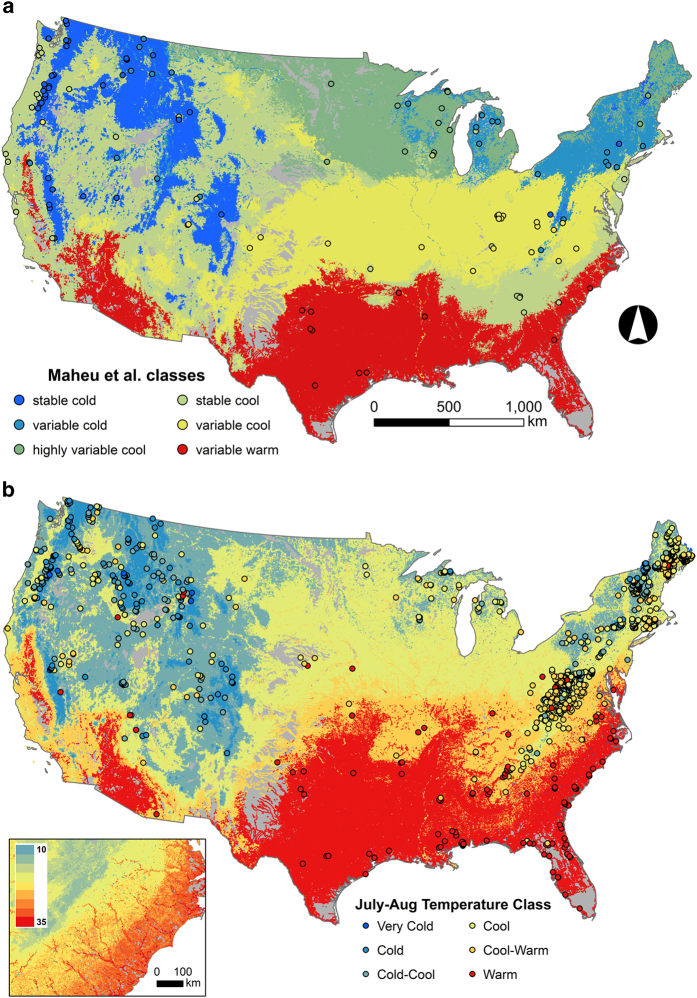
Temperature classes within stream reaches of the conterminous US. Temperature classes were mapped to stream reaches using (**a**) Maheu *et al.*^3^ thermal regime classes developed for stream gages (points), and (**b**) average July–August temperature values taken from multiple datasets (points). Inset in panel b shows the level of detail within continuous values of July–August temperature (°C) underlying the classification.

**Figure 7:**
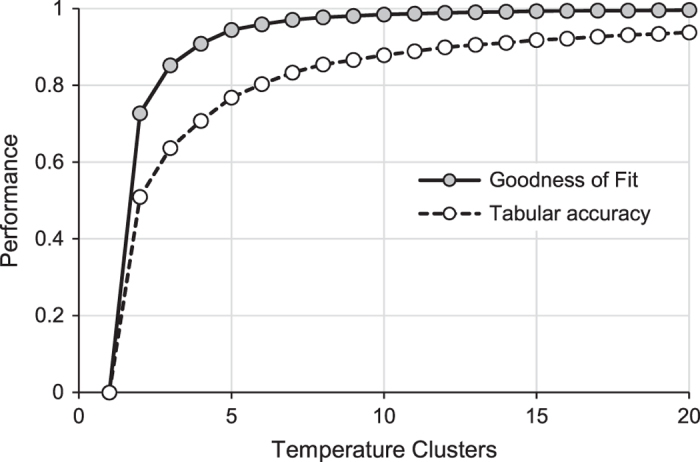
Thresholds for determining partitions between temperature classes. Goodness-of-fit and tabular accuracy for different numbers of temperature clusters using Jenks method.

The random forest model predicting Maheu *et al.* temperature classes had a 28% OOB misclassification rate (72% accuracy rate). For individual stream reaches, the median probability of predominant class membership was 0.45, compared to the expected probability of 0.17 (Table 7). Predominant Maheu *et al.* classes consisted of stable cool (27%), variable cool (25%), and variable-warm types (18%) (Fig. 6a, Supplementary File 2). Based on combinations of all sources, we identified 1764 reference sites across the CONUS, which were summarized into composite July–August temperatures for 1217 stream reaches (more than 1 station occurred in individual reaches). July–August water temperatures averaged 19.6°C and ranged from 7.08°C within a tributary of Salmon River near Snibnite, Idaho to 49.8°C within the Boiling River at Mammoth Yellowstone National Park, Wyoming. Random forest models predicting average July–August water temperatures explained 72% of variation with mean-squared error (MSE) of 4.60. Variables most important to predicting temperature classes and July–August temperature were associated with climate, but also a few basin characteristics (elevation, slope), vegetation land cover, and hydrology or hydrologic properties of soils (Fig. 4). Based on Jenks Natural Breaks method, goodness-of-fit and tabular accuracy reached a plateau at five classes indicating that 5 groups would be a parsimonious solution that also explained most of the variation in July–August temperatures (Fig. 7). Based on these class thresholds (Table 4), most reaches were classified as Cold (27%), Cool (24%), and Warm (24%) with rarest types being variable cold (8%) (Fig. 6b, Supplementary File 2).

**Figure 8:**
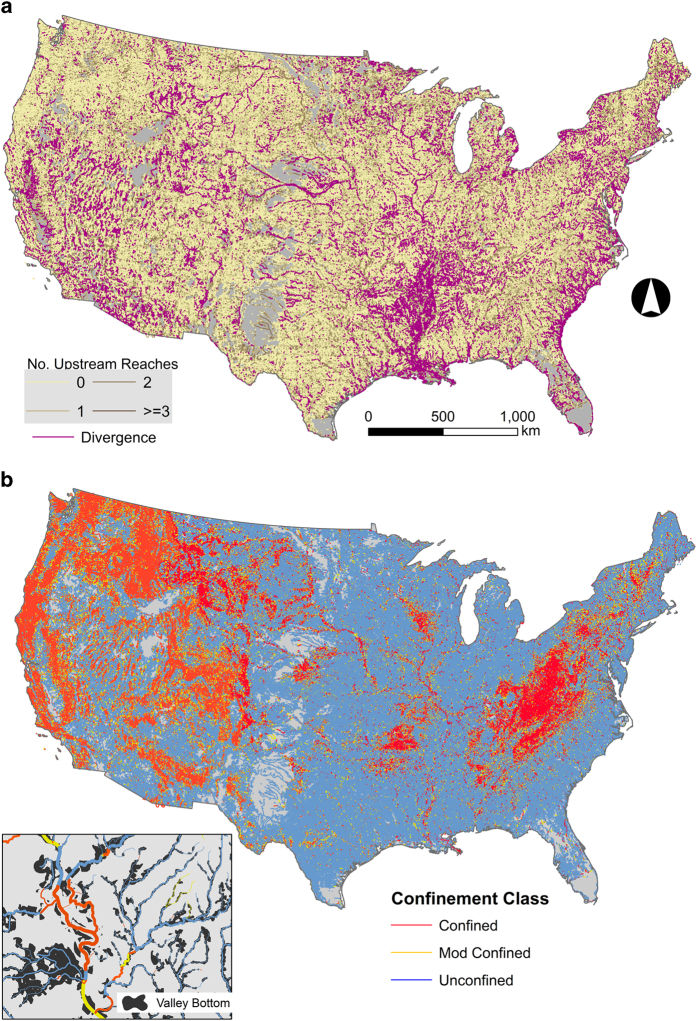
Network Bifurcation and valley confinement of stream reaches of the conterminous US. (**a**) Information used to develop network bifurcation classes in streams included the number of upstream reaches contributing to each reach and divergences in channels. Mapping bifurcation classes was impractical as there are 348 different types. (**b**) Valley confinement classes mapped to stream reaches. Inset provides example of valley bottoms underlaying streams of varying widths.

The assessment of network bifurcation yielded 348 classes representing unique combinations of stream order-tributary junctions. Of these, only 18 classes represented over 95% of the total stream length in the CONUS (Supplementary File 2). Almost 50% of total stream length was 1^st^ order streams without any upstream tributary confluence (i.e., 1_0), whereas less than 0.2% of stream length (<10,000 km) consisted of complex junctions, i.e. stream reaches formed by the confluence of three or more reaches (Fig. 8a, Supplementary File 2). Only 4 classes represented different types of divergence junctions. Stream reaches characterized as main or side-channel divergences by NHDplus V2 constituted 2% of total stream length (130,389 reaches, Fig. 8a) whereas stream reaches immediately downstream of divergences also comprised 2% of stream length (89,251 reaches) (Supplementary File 2). Additional stream reaches identified as divergence-type junctions (i.e., those having non-sensical junctions) totaled 5,376 reaches. Our estimates of bifurcation classes and associated sample sizes include correcting for non-meaningful stream junctions arising from quadrangle boundaries. A total of 133,111 stream reaches were flagged as being discretized into hydrologically unmeaningful segments^51^. We ensured all reaches belonging to a common ecological identifier unit were assigned the most upstream bifurcation class and divergence class.

Using the VCA tool, we identified over 1.2 million valley bottom floodplains constituting over 930,138 km^2^ in the CONUS. Characterizing valley confinement required comparing valley bottoms to estimates of river width. Based on >50,000 observations across the CONUS, river widths in the US ranged from <1 to 10,330 m and averaged 330 m. The random forest model explained 87.7% of variation in river width and had an MSE of 0.131. Hydrologic variables (estimated annual and monthly discharge) were the most important variables for predicting river widths (Fig. 4). Most stream reaches were classified as unconfined (64% of length), followed by confined (22%) and moderately confined (10%) reaches (Fig. 8b, Supplementary File 2). Stream reaches completely inundated by waterbodies constituted 3.4% of stream length (226,961 reaches) and could not be classified according to valley confinement. In cases where stream reaches were partially inundated, we used non-inundated sections to determine valley confinement status for the entire reach.

## Usage Notes

The SCS, in its entirety or specific layers therein, provides a geospatial data product useful to biogeographic applications (e.g., species distribution modeling), planning or prioritizing stream conservation and restoration activities, fluvial geomorphology research, or understanding the diversity of stream ecosystems for eventual representation in Earth System Models. Researchers and managers have varied reasons in using stream classifications; thus, we attempted to use alternative approaches in developing each layer, with preference for adopting previous published approaches at the scale of the entire US. Through several years of conversations with environmental stakeholders, we devised six principles that guided our classification and are aimed to maximize the use and application of the SCS product. Because the spatial framework of the SCS was devised using the NHDPlus V2 framework, the classes and associated attributes harness the utility imbedded within NHD products, such as the ability to traverse the stream network and conduct network accumulation and summarization of SCS attributes. Our data products include the NHDPlus V2 COMID, which is a common identifier that uniquely identifies each reach and provides an ability to join SCS data to the NHDPlus V2 dataset or datasets derived from that product.

As noted in the technical validation, using models to extrapolate classes or values from discrete *in situ* observations to stream reaches was prone to error; however, our reported error rates were well within the range of expected values based on similar analyses^6,34^. As much as possible, we provide information on uncertainty, such as probability of class membership, to support flexibility of use and allow users to account for uncertainty in subsequent analyses^6^. For instance, a reach may probabilistically share membership among multiple classes. These probabilities are useful for modeling, clustering streams, or identifying very rare or transitional stream types. Additionally, while we attempted to justify our approach to class partitioning, we acknowledge there are a multitude of approaches for partitioning stream classes. For example, users may desire to use alternative threshold values, such as those determined via biological discrimination, to modify the classification; hence, we also provide the variables behind the classification, where relevant, to support various uses.

For some layers, the number of classes may be overwhelming for a given application; however, our provision of class thresholds and class frequencies can help render simplified solutions. As stated previously, the size, gradient, and summer temperature classes can be coarsened based on values in Table 1. Likewise, the nested hierarchy of hydrologic classifications (i.e. Ward’s approach) provides flexibility in using coarser classes or sub-selecting nested groups. As another example, the network bifurcation effort yielded 348 combinations of stream-tributary orders; however, only 18 of the classes represented the vast majority (95%) of stream length in the US. Alternatively, stream divergences or the number of upstream or downstream tributaries could serve as simpler classifications.

Because layers within the SCS were developed using least-disturbance conditions, our classes and associated variables (e.g., average July–Aug temperature) inherently provide an indication of reference conditions or targets for mitigation. By comparing present-day conditions to values in the SCS, one can quickly determine the degree of habitat alteration for a given stream reach. Furthermore, combining multiple layers can provide a multi-dimensional characterization of stream ecosystems that can serve as a template for identifying reference sites to guide restoration^12^.

## Additional information

**How to cite this article**: McManamay, R. A. and DeRolph, C. R. A stream classification system for the conterminous United States. *Sci. Data*. 6:190017 https://doi.org/10.1038/sdata.2019.17 (2019).

**Publisher’s note**: Springer Nature remains neutral with regard to jurisdictional claims in published maps and institutional affiliations.

## Supplementary Material



Supplementary File 1

Supplementary File 2

Supplementary File 3

## Figures and Tables

**Table 1:** Datasets used in developing the US stream classification system.

Dataset	Theme	Description	Spatial Resolution	Temporal Resolution
NHDPlus V2^a^	Multiple	Hydrography of streamflow lines, waterbodies, and associated attributes summarized for local catchments at 1:100 k resolution	Stream Reaches, Catchments	NA
StreamCat^b^	Pred; Dist	Large suite of variables summarized in for NHDPlus V2 local catchments and upstream networks	Stream Reaches, Catchments	Decadal; Discrete Annual
WorldClim^c^	Pred	Bioclimatic variables summarized at seasonal periods from 30-yr normals (1970–2000)	1 km^2^	Decadal averages
USGS NWIS^d^	Hyd; Temp	Measurements of discharge (daily) and water temperature (daily and field grap samples) from ~1950 to 2017	Point	Daily; Grab Samples
StreamNet^e^	Temp	Daily water temperature data from deployable loggers (~1995–2000)	Point	Daily
Hill *et al.*^40^	Temp	July–August averages of daily water temperature (1999–2008)	Point	Seasonal
Deweber and Wagner^41^	Temp	July–August averages of daily water temperature (2002 to 2010)	Point	Seasonal
Wieferich *et al.*^51^	Bif	Common identifiers of reaches belonging to the same hydrologically meaningful unit. Identifiers are used to correct unmeaningful junctions, primarily resulting from intersections of reaches with quadrangle map boundaries	Stream Reaches	NA
NED 30-m^f^	ValC	National Elevation Dataset (30 m)	30 m grid	NA
NRSA^g^	ValC	US Environmental Protection Agency (EPA) National Rivers and Streams Assessment (NRSA). Provides river width in habitat assessments of sites.	Point	NA
Downing *et al.*^29^	ValC	Assemblage of river width information from published literature	Point	NA
NARWidth v0.1^h^	ValC	Landsat‐derived dataset of river widths in North America for >2.4 × 105 km of rivers wider than 30 m	River Segments	NA
PRISM 30 y Normals^i^	ValC	Gridded spatial datasets of average historical climate conditions, in this case, precipitation	800 m grid	30-year mean
NFHP^43^	Dist	National Fish Habitat Partnership assessment of habitat degradation within NHDPlus V1 catchments	Stream Catchments	NA
Pred = Predictor; Dist = Disturbance; Hyd = Hydrology; Temp = Temperature; Bif = Network Bifurcation; ValC = Valley Confinement ^a^NHDPlus V2: http://www.horizon-systems.com/nhdplus/NHDPlusV2_home.php. ^b^Environmental Protection Agency StreamCat: https://www.epa.gov/national-aquatic-resource-surveys/streamcat. ^c^WorldClim Global Climate Data: http://worldclim.org/. ^d^US Geological Survey National Water Information System: https://waterdata.usgs.gov/nwis. ^e^StreamNet: Fish Data for the Northwest: https://www.streamnet.org/. ^f^National Elevation Dataset (30-m): https://nationalmap.gov/elevation.html. ^g^National Rivers and Streams Assessment: https://www.epa.gov/national-aquatic-resource-surveys/nrsa. ^h^North American River Width Data Set v0.1: http://gaia.geosci.unc.edu/NARWidth/. ^i^PRISM 30-yr Normals: http://www.prism.oregonstate.edu/normals/.				

**Table 2:** Predictor variables and their sources assembled for random forest models.

Variable Name	Description	Source
**Basin Characteristics**		
WsAreaSqKm	Watershed area (square km) at NHDPlus stream segment outlet, i.e., at the most downstream location of the vector line segment	StreamCat
ElevWs	Mean watershed elevation (m)	StreamCat
StreamOrde	Modified Strahler Stream Order	NHDPlusV2
ArbolateSu	Arbolate Sum - Kilometers of stream upstream of the bottom of the NHDFlowline feature	NHDPlusV2
slopeAvgWs^∗^	Mean upstream channel slopes	NHDPlusV2
elevWsRng	Watershed range in elevation	NHDPlusV2
**Climate**		
PrecipWs	PRISM climate data - 30-year normal mean precipitation (mm): Annual period: 1981-2010 within the watershed	StreamCat
TminWs^∗^	PRISM climate data - 30-year normal minimum temperature (C°): Annual period: 1981-2010 within the watershed	StreamCat
TmeanWs	PRISM climate data - 30-year normal mean temperature (C°): Annual period: 1981-2010 within the watershed	StreamCat
TmaxWs	PRISM climate data - 30-year normal maximum temperature (C°): Annual period: 1981-2010 within the watershed	StreamCat
PrecipVC	Mean annual precipitation in area upstream of the bottom of flowline in millimeters ^∗^ 100 for the 1971 to 2000 time period	NHDPlusV2
TempVC	Mean annual temperature in area upstream of the bottom of flowline in degrees centigrade ^∗^ 100 for the 1971 to 2000 time period	NHDPlusV2
bcm10AvgWs	Mean temperature of warmest quarter	WorldClim
PET0001	Potential Evapotranspiration (mm) in catchment	NHDPlusV2
**Land Cover**		
PctWaterWs	% of watershed area classified as as lithology type: water	StreamCat
PctDecid2011Ws	% of watershed area classified as deciduous forest land cover (NLCD 2011 class 41)	StreamCat
PctConif2011Ws	% of watershed area classified as evergreen forest land cover (NLCD 2011 class 42)	StreamCat
PctMxFst2011Ws	% of watershed area classified as mixed deciduous/evergreen forest land cover (NLCD 2011 class 43)	StreamCat
PctOw2011Ws	% of watershed area classified as open water land cover (NLCD 2011 class 11)	StreamCat
PctIce2011Ws	% of watershed area classified as ice/snow land cover (NLCD 2011 class 12)	StreamCat
PctHbWet2011Ws	% of watershed area classified as herbaceous wetland land cover (NLCD 2011 class 95)	StreamCat
PctWdWet2011Ws	% of watershed area classified as woody wetland land cover (NLCD 2011 class 90)	StreamCat
PctShrb2011Ws	% of watershed area classified as shrub/scrub land cover (NLCD 2011 class 52)	StreamCat
PctGrs2011Ws	% of watershed area classified as grassland/herbaceous land cover (NLCD 2011 class 71)	StreamCat
**Soils/Geology**		
WtDepWs	Mean seasonal water table depth (cm) of soils (STATSGO) within watershed	StreamCat
OmWs	Mean organic matter content (% by weight) of soils (STATSGO) within watershed	StreamCat
PermWs	Mean permeability (cm/hour) of soils (STATSGO) within watershed	StreamCat
RckDepWs	Mean depth (cm) to bedrock of soils (STATSGO) within watershed	StreamCat
ClayWs	Mean % clay content of soils (STATSGO) within watershed	StreamCat
SandWs	Mean % sand content of soils (STATSGO) within watershed	StreamCat
PctCarbResidWs	% of watershed area classified as as lithology type: carbonate residual material	StreamCat
PctNonCarbResidWs	% of watershed area classified as as lithology type: non-carbonate residual material	StreamCat
PctAlkIntruVolWs	% of watershed area classified as as lithology type: alkaline intrusive volcanic rock	StreamCat
PctSilicicWs	% of watershed area classified as as lithology type: silicic residual material	StreamCat
PctExtruVolWs	% of watershed area classified as as lithology type: extrusive volcanic rock	StreamCat
PctColluvSedWs	% of watershed area classified as as lithology type: colluvial sediment	StreamCat
PctGlacTilClayWs	% of watershed area classified as as lithology type: glacial till, clayey	StreamCat
PctGlacTilLoamWs	% of watershed area classified as as lithology type: glacial till, loamy	StreamCat
PctGlacTilCrsWs	% of watershed area classified as as lithology type: glacial till, coarse-textured	StreamCat
PctGlacLakeCrsWs	% of watershed area classified as as lithology type: glacial outwash and glacial lake sediment, coarse-textured	StreamCat
PctGlacLakeFineWs	% of watershed area classified as as lithology type: glacial lake sediment, fine-textured	StreamCat
PctHydricWs	% of watershed area classified as as lithology type: hydric, peat and muck	StreamCat
PctEolCrsWs	% of watershed area classified as as lithology type: eolian sediment, coarse-textured (sand dunes)	StreamCat
PctEolFineWs	% of watershed area classified as as lithology type: eolian sediment, fine-textured (glacial loess)	StreamCat
PctSalLakeWs	% of watershed area classified as as lithology type: saline like sediment	StreamCat
PctAlluvCoastWs	% of watershed area classified as as lithology type: alluvium and fine-textured coastal zone sediment	StreamCat
PctCoastCrsWs	% of watershed area classified as as lithology type: coastal zone sediment, coarse-textured	StreamCat
KffactWs	The Kf factor is used in the Universal Soil Loss Equation (USLE) and represents a relative index of susceptibility of bare, cultivated soil to particle detachment and transport by rainfall within watershed	StreamCat
HydrlCondWs	Mean lithological hydraulic conductivity (micrometers per second) content in surface or near surface geology within watershed	StreamCat
CompStrgthWs	Mean lithological uniaxial compressive strength (megaPascals) content in surface or near surface geology within watershed	StreamCat
**Hydrology**		
RunoffWs	Mean runoff (mm) within watershed	StreamCat
BFIWs	Base flow is the component of streamflow that can be attributed to ground-water discharge into streams. The BFI is the ratio of base flow to total flow, expressed as a percentage, within watershed	StreamCat
Q0001A	Mean Annual Flow (cfs) estimated from Enhanced Runoff Method (EROM), applies to monthly averages below	NHDPlusV2
Q0001Ajan	Mean January flow (cfs) using EROM	NHDPlusV2
Q0001Afeb	Mean Febuary flow (cfs) using EROM	NHDPlusV2
Q0001Amar	Mean March flow (cfs) using EROM	NHDPlusV2
Q0001Aapr	Mean April flow (cfs) using EROM	NHDPlusV2
Q0001Amay	Mean May flow (cfs) using EROM	NHDPlusV2
Q0001Ajun	Mean June flow (cfs) using EROM	NHDPlusV2
Q0001Ajul	Mean July flow (cfs) using EROM	NHDPlusV2
Q0001Aaug	Mean August flow (cfs) using EROM	NHDPlusV2
Q0001Asep	Mean September flow (cfs) using EROM	NHDPlusV2
Q0001Aoct	Mean October flow (cfs) using EROM	NHDPlusV2
Q0001Anov	Mean November flow (cfs) using EROM	NHDPlusV2
Q0001Adec	Mean December flow (cfs) using EROM	NHDPlusV2
Qwsa	Q0001A divided by WsAreaSqKm	This study
For information on sources, please see footnotes of Table 1.		

**Table 3:** Datasets provided by the US Stream Classification System.

Dataset	Classification Variables	Continuous/Nominal Variables
Size and Gradient	Size class; Gradient class	Strahler Stream Order, Mean Annual Discharge; Stream-reach slope
Hydrology	Hydrologic classes (Bayesian Gaussian Mixed Model and Ward’s Agglomerative Hydrologic classes)	Probabilities of class assignment from random forests
Temperature	Temperature Classes (Maheu *et al.* classes and average July–August temperature classes)	Probabilities of class assignment from random forests; Predicted July–August water temperature
Bifurcation network	Bifurcation Classes, Divergence Classes	Upstream Reach Count; Downstream reach count; Upstream orders; ecological unit identifier; flagged reaches (for unmeaningful junctions)
Valley Confinement	Valley confinement classes	Stream reach length (RL); catchment area; river width area (RWA); valley bottom area (VBA); valley bottom length (VBL); VBL:RL ratio; VBA:RWA ratio

**Table 4:** Thresholds used to partition classes based on univariate continuous data.

Size	Range (m^3^s^−1^)	Gradient	Range (Rise/Run)	Avg Summer Temperature	Range (°C)
Headwater (HW)	0–0.057	Very Low (VL)	<0.001	Very Cold (VC)	<10
Small Creek (SC)	0.057–0.283	Low (L)	0.001–0.005	Cold (CD)	10–15
Large Creek (LC)	0.283–1.133	Moderate (M)	0.005–0.02	Cold-Cool (CC)	15–18
Small River (SR)	1.133–5.663	Moderate High (MH)	0.02–0.04	Cool (CL)	18–21
Medium River (MR)	5.663–22.65	High (H)	0.04–0.1	Cool-Warm (CW)	21–24
Mainstem (MS)	22.65–70.79	Steep (S)	>0.1	Warm (W)	>24
Large River (LR)	70.79–283.2	….	….	….	….
Great River (GR)	>283.2	….	….	….	….

**Table 5:** Gaussian mixed model hydrologic class names and their codes.

Mixture Model Classes	Abbr. Code	Num. Code
Intermittent Flashy 1	IF1	1
Late Timing Runoff	LTR	2
Perennial Runoff 1	PR1	3
Perennial Runoff 2	PR2	4
Super Stable Groundwater	SSGW	5
Stable High Baseflow	SHBF	6
Intermittent Flashy SW	IFSW	7
Snowmelt 2	SNM2	8
Perennial Flashy	PF	9
Intermittent Flashy 2	IF2	10
Western Coastal Runoff	WCR	11
Stable High Runoff	SHR	12
Harsh Intermittent	HI	13
Snowmelt 1	SNM1	14
Glacial High Runoff	GHR	15
Names and their geographic and hydrologic descriptions are provided by McManamay *et al.*^34^.		

**Table 6:** Nested hierarchy of hydrologic classes developed using Ward’s agglomerative method.

2 clusters	4 clusters	8 clusters	14 clusters	30 clusters
1. Low Baseflow (LBF)	1. Perennial (P)	1. Perennial Runoff (PR)	1. Perennial Runoff 1 (PR1)	1. Perennial Runoff 1 N (PR1N)
				4. Perennial Runoff 1 S (PR1S)
			3. Perennial Runoff 2 (PR2)	3. Perennial Runoff 2 W (PR2W)
				5. Perennial Runoff 2 E (PR2E)
		4. Perennial Flashy (PF)	5. Perennial Flashy (PF)	7. Perennial Flashy 1 (PF1)
				11. Perennial Flashy 2 (PF2)
				30. Perennial Flashy 3 (PF3)
		8. Western Runoff (WR)	14. Western Runoff (WR)	29. Western Runoff 1 (WR1)
				28. Western Runoff 2 (WR2)
	4. Intermittent (I)	5. Intermittent (I)	7. Unpredictable Intermittent (UI)	19. Unpredictable Intermittent 1 (UI1)
				9. Unpredictable Intermittent 2 (UI2)
			8. Late Timing Intermittent (LTI)	10. Late Timing Intermittent 1 (LTI1)
				22. Late Timing Intermittent 2 (LTI2)
			10. Intermittent Flashy 1 (IF1)	14. Intermittent Flashy 1 (IF1)
			12. Intermittent Flashy 2 (IF2)	17. Intermittent Flashy 2 A (IF2A)
			21. Intermittent Flashy 2B (IF2B)	
		7. Intermittent SW (ISW)	13. Intermittent SW (ISW)	24. Intermittent SW A (IFSWA)
				26. Intermittent SW B (IFSWB)
				18. Intermittent SW C (IFSWC)
				23. Intermittent SW D (IFSWD)
2. High Baseflow (HBF)	2. Snowmelt (SNM)	2. Snowmelt 1 (SNM1)	2. Snowmelt 1 (SNM1)	2. Snowmelt 1 A (SNM1A)
				25. Snowmelt 1B (SNM1B)
		6. Snowmelt 2 (SNM2)	11. Snowmelt 2 (SNM2)	16. Snowmelt 2 A (SNM2A)
				20. Snowmelt 2B (SNM2B)
				15. Glacial Snowmelt (GSNM)
	3. Stable Baseflow (SBF)	3. Stable Baseflow (SBF)	4. Stable High Baseflow (SHBF)	6. Stable High Baseflow (SHBF)
				27. Western Stable High Baseflow (WSHBF)
			6. Super Stable GW 1 (SSGW1)	8. Super Stable GW 1 (SSGW1)
			9. Super Stable GW 2 (SSGW2)	12. Super Stable GW 2A (SSGW2A)
				13. Super Stable GW 2B (SSGW2B)
Clustering was based on Principal Components reducing the dimensionality of hydrologic statistics summarized for discharge among 2512 US Geological Survey stream gages.				

**Table 7:** Accuracies, cross-validation error rates, and propabilities for random forest models predicting hydrologic and temperature classes.

Cluster approach	Theme	Classes (clusters)	N obs	OOB error rate (%)	Classification accuracy (%)	Median Prob.
Gaussian Mixture Model Bayesian	HYDR	15	2512	23.2	76.8	0.43 (0.07)
Ward’s agglomerative	HYDR	2	2512	4.94	95.1	0.91 (0.50)
Ward’s agglomerative	HYDR	4	2512	9.12	90.9	0.73 (0.25)
Ward’s agglomerative	HYDR	8	2512	17.0	83.0	0.58 (0.13)
Ward’s agglomerative	HYDR	14	2512	24.6	75.4	0.46 (0.07)
Ward’s agglomerative	HYDR	30	2512	33.8	66.2	0.34 (0.03)
Maheu *et al.* 2016	TEMP	6	135	28.2	71.9	0.45 (0.17)
OOB = out-of-bag error rate, i.e. cross validation error rate. Median Prob. = Median probability of predominant class assignment to all reaches compared to expected probabilities (in parentheses).						

## References

[d1] figshare McManamay R. A. DeRolph C. R. 2018 10.6084/m9.figshare.c.4233740

