# Supplementary material for: A stream classification system for the conterminous United States
Source: Sci Data. 2019 Feb 12;6:190017. doi: 10.1038/sdata.2019.17 (PMC6371895; doi:10.1038/sdata.2019.17)
Supplement: Supplementary File 1 [file sdata201917-s2.pdf]

Supplementary File 1. Record length determination for adequate estimation of average July-August water temperatures

A Stream Classification System for the Conterminous United States

Ryan A. McManamay and Christopher R. DeRolph

Environmental Sciences Division, Oak Ridge National Laboratory, Oak Ridge, TN 37831

a Corresponding author:
mcmanamayra@ornl.gov
One Bethel Valley Rd.
P.O. Box 2008, MS-6351
Oak Ridge, TN 37831-6351
865-241-8668

Jones and Schmidt (ref. 42 main paper) provide an assessment of uncertainty of 9 thermal metrics depending on the record length used to derive those metrics. Of these mean July daily temperature range best approximates average July-August temperatures in our study. Based on their assessment, mean July daily temperature range can be accurately estimated within 1°C with 95% confidence using 7.4 years of temperature data, 90% confidence using 6 years of data, with 75% confidence using 3.7 years of data. However, mean July daily temperature range does not necessarily approximate a bi-monthly average for summer temperatures used in our study. Therefore, we examined year-to-year variation in July and August temperature ranges versus year-to-year variation in July and August means at 22 USGS gages across the US (Table S1-1). On average, standard deviation (sd) in July and August ranges was 1.34 and 1.55 times the sd in July and August means. Additionally, range sd could be as high as 4 times that of monthly mean sd (Table S1-1).

To extend Jones and Schmidt's findings to our study, we first had to estimate sd in mean July daily temperature range for their samples. Cumulative probabilities, sample size, and years required to obtain those cumulative probabilities were provided by Jones and Schmidt (Table S1-2). For each cumulative probability, we determined the Z statistic and subsequently the sd (σ) using the following formula:

$$\sigma = \frac{(CI)}{Z} \times \sqrt{n}$$

Where CI is the confidence interval and n is the sample size, in this case 18.

Based on our assessment of 22 USGS gages, year-to-year σ in ranges of July and August temperatures are approximately 1.5 times the year-to-year σ in averages of July and August temperatures Thus, we divided σ values in Table S2-2 by 1.5 to derive estimates of σ applicable to Jones and Schmidt's study, but also relevant to July-August averages (Table S1-3). These σ values along with CI and sample sizes were used to calculate new Z statistics and associated confidence bands (Table S1-3). This estimates that with 3.7 years of data, estimates of July-August averages can be reliable at 1°C with 93.5% confidence (Table S1-3). However, to match recommendations of Jones and Schmidt, we need to extend these values to comparable confidence bands, i.e., 75%, 90%, and 95%. To do so, we developed an exponential relationship between the desired confidence level and the number of years required to obtain that confidence level (Figure S1-1). Using this relationship, we extrapolated estimates of years required to obtain 75%, 80%, 90%, and 95% confidence levels.

References:

Jones, N.E., & Schmidt, B.J. Thermal regime metrics and quantifying their uncertainty for North American streams. River Res Appl. **34**, 382-393 (2018).

Table S1-1. US Geological Gages used to compare year-to-year variation in July and August mean water temperatures to year-to-year variation in the range of July and August values. σ represents standard deviation across all years. σ Ratio represents σ Range divided by σ Mean.

			July			August		
**Gage ID**	**Name**	**Years**	**σ Mean**	**σ Range**	**σ Ratio**	**σ Mean**	**σ Range**	**σ Ratio**
**01115190**	Dolly Cole Bk at Old Danielson Pk at S Foster, RI	12	1.34	1.44	1.08	1.01	2.06	2.05
**01467861**	West Cr West Branch Schuylkill near Pottsville, PA	4	1.11	0.45	0.41	0.89	0.35	0.40
**02135000**	Little Pee Dee River at Galivants Ferry, SC	6	1.60	1.73	1.08	1.60	1.86	1.17
**02323592**	Suwannee River ab Gopher River near Suwannee FL	14	1.10	0.59	0.54	1.21	0.89	0.74
**02330450**	Chattahoochee River at Helen, GA	9	1.21	0.73	0.60	1.42	0.62	0.44
**02480285**	West Pascagoula River at Hwy 90 at Gautier, MS	12	0.79	3.27	4.16	0.77	3.56	4.65
**03011020**	Allegheny River at Salamanca NY	5	2.33	2.69	1.15	1.79	0.61	0.34
**03538830**	Obed River at Adams Bridge near Crossville, TN	3	0.29	0.69	2.37	0.47	0.85	1.82
**04043150**	Silver River near L'anse, MI	10	1.78	2.15	1.21	1.21	1.68	1.39
**06192500**	Yellowstone River near Livingston, MT	14	1.55	1.29	0.83	0.57	1.40	2.47
**06796550**	Platte River near Venice, NE	6	1.33	3.30	2.48	0.91	1.77	1.95
**06879650**	Kings C near Manhattan, KS	3	0.57	0.66	1.15	1.12	1.41	1.27
**07227500**	Canadian Rv near Amarillo, TX	21	0.86	1.93	2.24	1.21	2.04	1.69
**08227000**	Saguache Creek near Saguache, CO	4	1.11	0.30	0.27	0.71	0.83	1.17
**09471000**	San Pedro River at Charleston, AZ	5	1.02	0.69	0.67	0.93	0.80	0.86
**09505800**	West Clear Creek near Camp Verde, AZ	4	0.52	0.89	1.73	0.81	1.19	1.47
**10249300**	S Twin Rv near Round Mountain, NV	9	1.02	1.44	1.42	0.74	0.57	0.77
**10336770**	Trout Ck At Usfs Rd 12n01 near Meyers, CA	6	0.61	0.86	1.42	0.50	1.07	2.14
**11501000**	Sprague River near Chiloquin, OR	12	0.69	0.98	1.41	0.42	0.94	2.22
**12340000**	Blackfoot River near Bonner MT	17	1.20	1.22	1.02	0.60	1.33	2.23
**12355000**	Flathead River at Flathead British Columbia	15	1.03	1.33	1.29	0.67	1.06	1.58
**13069500**	Snake River near Blackfoot ID	17	1.26	1.25	0.99	0.91	1.08	1.19
	**Total Average**				**1.34**			**1.55**

Table S1-2. Number of years required to estimate the range in July temperatures within 1°C, dependent upon different confidence bands (i.e., cumulative probabilities), along with other population statistics (from Jones and Schmidt 2018). Estimates of standard deviation (σ) were calculated for different confidence bands based on Z statistics. Z statistics were obtained from Z table for each confidence band.

Cumulative Prob.	Z statistic	N	Confidence Interval (°C)	Calculated σ	Yrs Required
0.75	1.064	18	1	3.987	3.7
0.90	1.734	18	1	2.447	6.0
0.95	2.101	18	1	2.019	7.4

Table S1-3. Number of years required to estimate average July and August temperatures within 1°C, dependent upon different confidence bands (i.e., cumulative probabilities). We estimated Z statistics (and associated confidence bands) based on reduced standard deviation (σ) values. Assuming σ for range in July values (Table S2-2) is 1.5 times the σ for average values in July and August, we divided values for σ reported in Table S2-2 by 1.5. Again, Z statistics were obtained from Z table for each confidence band.

Calculated Cumulative Prob.	Calculated Z statistic	N	Confidence Interval (°C)	σ est.	Yrs Required
0.94	1.596	18	1	2.658	3.7
0.99	2.601	18	1	1.631	6.0
1.00	3.152	18	1	1.346	7.4

Table S1-4. Estimates of the number of years required to accurately estimate average July and August temperatures within 1°C, dependent upon different confidence bands (i.e., cumulative probabilities). Using data from S2-3, we developed an exponential relationship between the desired confidence level and the number of years required to obtain that confidence level (see Figure S2-1).

Confidence Band	Estimated Years Required
0.75	0.5
0.80	0.9
0.90	2.5
0.95	4.3

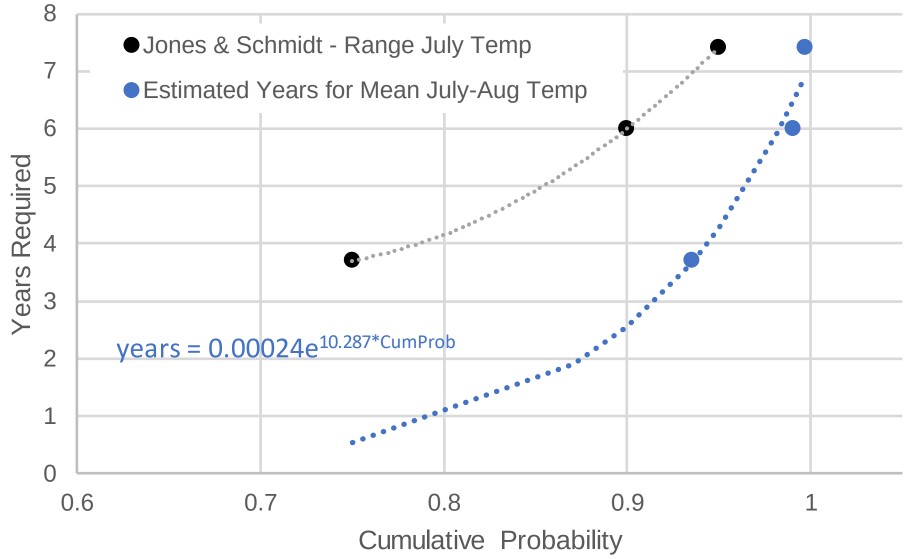

Figure S1-1. Exponential relationship between the desired confidence level and the number of years required to obtain that confidence level for range in July temperatures (from Jones and Schmidt 2018) and mean July-August temperatures (this study)
