# Supplementary material for: A stream classification system for the conterminous United States
Source: Sci Data. 2019 Feb 12;6:190017. doi: 10.1038/sdata.2019.17 (PMC6371895; doi:10.1038/sdata.2019.17)
Supplement: Supplementary File 2 [file sdata201917-s3.pdf]

Supplementary File 2. Class frequencies and stream length summarization

A Stream Classification System for the Conterminous United States

Ryan A. McManamay and Christopher R. DeRolph

Environmental Sciences Division, Oak Ridge National Laboratory, Oak Ridge, TN 37831

a Corresponding author:
mcmanamayra@ornl.gov
One Bethel Valley Rd.
P.O. Box 2008, MS-6351
Oak Ridge, TN 37831-6351
865-241-8668

The tables below represent the frequency of stream reaches and sum of stream lengths according to each class.

Table S2-1. Stream Order Classes

Stream Order	Count	Length (km)
1	1335626	3079672
2	594826	975428
3	318361	524302
4	179817	280625
5	100262	147872
6	56302	78104
0	67032	54428
7	26751	34475
8	9479	13638
9	3033	5018

Table S2-2. Size classes determined from discharge.

Size Classes	Count	Length (km)
HW	1481546	2947149
SC	542257	1139800
LC	261629	502085
SR	169653	289972
MR	84392	132600
MS	41830	61607
LR	28388	40330
GR	15353	27820
None	67032	54428

Table S2-3. Gradient classes.

Gradient Class	Count	Length (km)
Moderate	823189	1761375
Low	563238	1179922
Very low	639290	764416
Moderate High	282383	623130
High	226187	547078
Steep	134168	275790
Blank	21257	37467

Table S2-4. Gaussian Mixture Model classes

Hydrologic Classes	Count	Length
IF2	307890	759124
SSGW	290594	619047
PR1	359183	586495
IF1	246219	582713
PF	276181	511531
LTR	224290	437416
SHBF	189903	297408
SNM2	162170	293218
PR2	174133	290874
SNM1	112930	209350
WCR	103967	199010
IFSW	82609	185499
HI	28432	89709
SHR	31784	66300
GHR	4049	5978
None	97746	62118

Table S2-5. Ward's agglomerative classes (two clusters)

Hydrologic classes	Count	Length
LBF	2111248	4207145
HBF	483086	926528
None	97746	62118

Table S2-6. Ward's agglomerative classes (four clusters)

Hydrologic classes	Count	Length
I	987899	2265688
P	1098439	1893976
SBF	246032	491150
SNM	261964	482859
None	97746	62118

Table S2-7. Ward's agglomerative classes (eight clusters)

Hydrologic classes	Count	Length
I	830641	1937568
PR	616793	1004386
PF	419084	758199
SBF	295262	604645
SNM1	171395	317255
ISW	127743	260440
SNM2	78733	139661
WR	54683	111519
None	97746	62118

Table S2-8. Ward's agglomerative classes (fourteen clusters)

Hydrologic classes	Count	Length
PF	477160	861696
PR2	386005	616193
UI	251894	550141
IF2	196906	530708
SNM1	216777	411079
PR1	220928	376619
ISW	172435	374521
SSGW2	171461	365240
LTI	165374	353790
IF1	133819	320950
SNM2	82284	145709
WR	56282	114610
SSGW1	34645	67073
SHBF	28364	45343
None	97746	62118

Table S2-9. Ward's agglomerative classes (thirty clusters)

Hydrologic classes	Count	Length
PF2	250915	457913
PF1	211614	374271
IF1	143579	338589
PR2E	198412	323707
PR1S	185026	308109
IF2A	118206	290602
LTI1	140348	279397
UI1	130320	278084
PR2W	169134	274041
SSGW2A	110841	266923
UI2	105484	242457
IFSWA	113862	240146
IF2B	76694	229070
IFSWC	70928	152464
PR1N	81675	145185
SNM1A	82821	141034
SSGW1	62351	127638
SNM2A	69709	122292
SNM1B	49487	95549
SSGW2B	53988	94468
LTI2	27398	75685
WR2	34108	65439
IFSWD	25410	60378
WR1	23017	50330
SHBF	31281	49642
IFSWB	16084	29965
SNM2B	7093	13452
GSNM	2523	3970
WSHBF	2026	2872
None	97746	62118

Table S2-10. Maheu et al. temperature classes

Temperature classes	Count	Length
stable cool	731333	1491426
variable cool	676101	1300765
var_warm	479705	948706
highly variable cool	261337	526575
stable cold	263725	517173
variable cold	209839	360234
None	70040	50911

Table S2-11. July-August temperature classes

Temperature classes	Count	Length
Cold	654896	1411534
Cool	629024	1255231
Warm	650670	1221796
Cool-Warm	533084	969479
Very Cold	141324	280605
None	83082	57146

Table S2-12. Divergence classes

Divergence Class	Count	Length (km)
None - No divergence	2467064	4989126
D - Divergence Unknown	5376	5410
D1 - Divergence Main Channel	65032	51047
D2 - Divergence Secondary Channel	65357	55648
DU - Upstream divergence	89251	94559

Table S2-14. Bifurcation classes

Bifurcation Classes	Count	Length
1_0	934644	2521838.48
1_1	405713	549823.128
2_1.2	239538	430434.022
2_1.1	226071	398058.438
3_1.3	141167	248414.74
2_2	122174	128914.177
4_1.4	73167	125686.997
3_2.3	54642	100170.675
3_2.2	50756	93728.343
3_3	63704	66615.942
5_1.5	34119	57750.909
4_2.4	32089	57066.594
4_4	42357	40194.96
5_2.5	17116	30834.602
5_5	31449	26790.619
6_1.6	15267	25352.265
0_0	15467	25066.629
4_3.4	12694	23492.229
4_3.3	11289	21554.093
6_2.6	9169	18025.792
2_2.2	11078	16358.483
6_6	23067	15756.035
3_3.3	10069	13776.152
5_3.5	7223	13691.793
4_4.4	9888	11260.195
7_1.7	5903	9912.484
7_7	13432	9653.068
1_1.1	6972	8134.892
5_5.5	7331	8048.472
6_3.6	4144	7951.137
0_1.1	3292	7741.334
7_2.7	3378	6106.542
5_4.5	2884	5664.253
6_6.6	5475	4884.182
5_4.4	2484	4813.497
8_1.8	2310	3797.722
8_8	4399	3654.599
6_4.6	1793	3617.812
7_3.7	1791	3203.157

0_2.2	1457	3118.983
7_7.7	3062	2825.266
8_2.8	1409	2552.93
1_1.2	1766	2169.93
1_1.3	1487	1689.048
9_1.9	983	1631.703
7_4.7	810	1565.095
2_1.2.1	934	1506.541
6_5.6	698	1430.8
8_3.8	744	1404.959
1_1.4	1349	1399.088
6_5.5	532	1199.547
9_2.9	646	1154.97
2_1.1.1	675	1084.222
1_1.5	941	1017.681
3_1.3.1	600	962.507
8_8.8	814	930.323
0_3.3	557	913.896
9_9	983	901.284
10_1	276	872.58
2_2.3	708	831.478
3_2.2.2	472	802.588
8_4.8	341	744.763
7_5.7	328	731.004
10_1.1	152	720.634
2_2.4	710	709.307
4_1.4.1	391	641.31
9_3.9	340	613.838
3_1.2.3	345	605.171
1_1.6	626	594.89
2_2.5	521	499.824
0_4.4	249	393.829
4_1.2.4	228	376.533
3_3.4	335	350.175
1_1.7	351	343.011
9_4.9	148	329.755
8_5.8	170	318.001
7_6.7	134	312.233
2_2.6	339	308.344
10_1.2	82	304.302
4_3.3.3	149	289.849
3_3.5	268	261.169
7_6.6	116	241.283

5_1.5.1	122	204.595
2_2.7	221	204.374
5_1.2.5	119	203.413
4_1.4.4	97	192.359
9_9.9	115	191.424
3_1.3.3	99	170.325
3_2.3.2	104	162.863
4_4.4.4	145	160.15
3_3.7	159	156.082
9_5.9	71	153.041
3_3.3.3	117	141.564
10_1.3	37	135.98
3_3.6	172	135.368
8_6.8	66	130.703
4_2.4.2	81	127.588
4_4.5	110	120.481
4_4.6	101	111.506
4_1.3.4	76	111.395
5_5.5.5	107	109.788
0_5.5	108	107.285
2_2.8	102	106.386
2_2.2.2	98	104.972
6_1.6.1	53	89.658
1_1.8	122	85.879
5_2.5.2	47	84.563
0_6.6	66	79.03
10_1.5	17	78.887
5_1.3.5	45	72.775
6_6.6.6	63	71.05
5_1.5.5	40	70.951
10_1.4	24	68.109
9_6.9	31	67.126
6_1.2.6	40	66.583
8_7.8	26	65.44
5_4.4.4	34	64.167
4_2.4.4	16	58.081
8_1.2.8	20	54.86
5_2.5.5	15	53.954
4_4.7	47	52.211
6_2.6.2	18	50.999
4_2.3.4	27	49.265
6_1.6.6	22	47.528
2_0	38	46.742

7_1.7.1	31	41.947
4_3.4.3	24	41.925
8_7.7	23	41.25
8_1.8.1	28	39.351
0_7.7	38	35.758
5_1.4.5	18	35.46
3_3.8	49	32.931
9_7.9	8	32.189
10_1.7	4	31.104
7_1.7.7	15	28.006
3_2.2.5.5	1	26.688
1_1.1.1	31	26.537
5_3.4.5	8	26.532
6_6.5.6	4	26.099
7_2.7.2	10	25.019
1_1.9	25	24.9
3_0	17	23.25
4_4.8	27	22.05
5_3.5.3	17	21.133
8_2.4.8	8	20.893
2_1.2.3	9	20.121
5_2.3.5	11	19.917
7_1.3.7	10	19.67
6_2.3.6	13	19.67
2_1.2.4	10	18.92
2_1.4.1	11	18.884
7_2.7.7	5	18.528
8_2.3.8	7	18.479
3_3.3.1	12	18.387
7_1.2.7	13	18.019
9_3.6	3	16.742
5_5.7	18	16.453
2_2.9	17	16.177
7_5.7.5	3	16.056
6_6.7	13	15.88
5_5.6	29	15.756
6_1.3.6	10	15.712
2_1.3.1	14	15.478
4_0	11	15.28
9_1.3.9	4	14.807
2_2.2.1	12	14.562
4_3.3.3.3	4	14.424
9_8.9	6	14.059

8_2.8.2	7	14.029
5_5.8	15	13.841
8_1.3.8	7	13.447
9_1.5	14	13.425
2_3.3.3	7	13.007
0_2.2.2	3	12.775
5_2.4.5	9	12.683
7_7.7.7	26	12.438
3_3.9	11	12.337
0_1.1.1	7	12.051
1_3.3.3	9	11.292
10_1.6	5	10.84
6_5.5.5	5	10.829
9_3.4.9	4	10.687
8_8.8.8	8	10.339
7_1.4.7	6	10.205
6_6.8	5	10.064
4_1.1.2.4	3	9.81
2_1.5.1	9	9.585
0_1.2.1	3	9.566
0_1.3.1	2	9.466
9_1.6	6	9.416
9_2.4.9	2	9.398
1_1.3.4	2	8.391
2_1.2.5	5	8.309
6_2.6.6	3	8.308
7_3.7.3	4	8.246
0_3.4.3	1	8.015
9_1.2.9	4	8.003
4_1.2.3.4	3	7.976
3_2.2.2.2	3	7.887
6_3.6.3	4	7.771
3_2.3.4	5	7.625
7_2.3.7	4	7.62
9_8.8	6	7.499
8_4.8.4	1	7.291
2_1.7.1	2	7.033
9_1.4.9	1	6.641
4_4.2.4	7	6.583
3_1.1.1.3	1	6.456
1_2.2.2	9	6.225
8_7.7.7	1	6.079
9_2.9.2	2	5.915

3_3.2.3	5	5.908
3_1.3.4	3	5.846
6_1.5.6	4	5.675
5_4.4.4.4	3	5.674
8_1.4.8	3	5.645
6_2.4.6	3	5.497
0_2.3.2	1	5.396
5_5.4.5	3	5.124
7_3.5.7	1	5.097
7_3.4.7	1	5.084
9_6	5	4.974
3_2.2.2.3	1	4.584
5_4.5.4	4	4.547
2_4.4.4	4	4.496
3_2.5.2	2	4.463
9_2.5	3	4.378
9_2.3.9	2	4.313
1_4.4.4	4	4.19
1_1.4.5	1	4.139
4_2.2.2.4	1	4.113
10_1.9	1	3.726
2_1.7.7	2	3.646
1_1.2.1	6	3.624
4_1.1.1.4	2	3.57
3_2	1	3.46
2_6.6.6	3	3.424
8_1.5.8	2	3.336
2_1.1.1.1	2	3.245
1_1.5.1	4	3.226
3_2.4.2	2	3.198
0_1	4	3.119
4_3.4.5	1	3.104
7_7.8	2	3.023
9_5.5	1	3.017
6_3.6.6	2	2.933
8_1.6.8	1	2.907
1_1.2.3	1	2.837
1_6.6.6	2	2.769
0_9.9	1	2.721
6_1.4.6	4	2.681
9_1.3.5	1	2.675
9_2.4	2	2.649
1_5.5.5	3	2.59

6_4.6.4	1	2.508
9_2.3	1	2.506
9_3.5	3	2.506
9_3.3	1	2.499
9_4.5	1	2.49
2_7.7.7	3	2.413
8_3.4.8	2	2.34
7_1.1.2.7	1	2.329
9_4.6	2	2.295
8_1.1.1.8	1	2.239
10_1.8	1	2.121
2_1.6.1	2	2.066
2_5.5.5	3	2.059
5_5.3.5	2	2.049
4_4.4.1	2	1.94
9_2.6	2	1.851
6_3.4.6	2	1.822
2_1.1.1.2	1	1.812
3_2.7.2	1	1.797
6_2.2.2.6	1	1.769
6_2.5.6	2	1.748
4_3.7.3	1	1.693
3_3.3.2	2	1.618
8_3.8.3	1	1.591
4_4.4.4.4	1	1.571
3_6.6.6	3	1.56
9_4.4	1	1.527
9_9.9.9	2	1.444
9_3	2	1.424
0_1.6.1	1	1.421
9_3.4.5.9	1	1.41
2_1.2.6	2	1.401
7_6.6.6	1	1.335
9_6.6	1	1.329
9_7	1	1.323
7_2.6.7	2	1.322
1_1.4.1	2	1.287
3_3.4.3	1	1.271
9_4	1	1.235
2_1.2.7	2	1.206
3_1.3.7	4	1.162
8_6.8.6	1	1.094
5_3.5.5	1	1.073

9_1.7.9	1	1.072
5_5.9	3	1.052
0_8.8	4	1.042
3_7.7.7	3	1.037
1_1.2.5	1	1.036
9_3.9.3	1	1.019
4_4.9	1	0.983
9_3.4	1	0.968
1_1.6.1	1	0.925
4_2.4.5	1	0.914
1_7.7.7	2	0.902
9_0	1	0.797
2_1.3.3	1	0.758
3_3.3.3.3	1	0.757
7_3.7.7	1	0.728
2_1.6.6	2	0.703
10_9.9	1	0.69
3_2.3.5	1	0.647
5_1.1.1.5	1	0.635
8_2.6.8	1	0.614
2_1.4.4	1	0.599
6_1.6.7	1	0.597
4_2.4.7	1	0.574
3_1.3.6	1	0.556
5_3.3.3	1	0.495
7_0	4	0.49
7_2.5.7	1	0.48
2_2.6.2	1	0.476
7_2.4.7	1	0.42
0_3.3.3	1	0.38
1_1.2.6	2	0.352
4_4.5.4	1	0.341
6_5.6.5	1	0.324
6_3.3.3	1	0.305
4_2.4.6	1	0.304
0_2.8.2	1	0.301
10_1.1.3	1	0.29
9_2.6.9	1	0.284
3_3.5.7	1	0.283
1_8.8.8	1	0.24
10_1.4.6	1	0.21
9_1.9.1	2	0.206
10_1.1.1	1	0.18

2_2.3.2	1	0.162
3_4.4.4	2	0.158
6_1.1.1.6	1	0.105
1_1.1.1.1	1	0.095
0_2.4.2	1	0.079
3_1.3.5	1	0.06
7_9	1	0.018
8_3.3.6.8	1	0
