# Supplementary material for: A stream classification system for the conterminous United States
Source: Sci Data. 2019 Feb 12;6:190017. doi: 10.1038/sdata.2019.17 (PMC6371895; doi:10.1038/sdata.2019.17)
Supplement: Supplementary File 3 [file sdata201917-s4.pdf]

Supplementary File 3. Dendrograms for Ward’s Agglomerative Clustering of Hydrologic Classes

A Stream Classification System for the Conterminous United States

Ryan A. McManamay and Christopher R. DeRolph

Environmental Sciences Division, Oak Ridge National Laboratory, Oak Ridge, TN 37831

a Corresponding author:
mcmanamayra@ornl.gov
One Bethel Valley Rd.
P.O. Box 2008, MS-6351
Oak Ridge, TN 37831-6351
865-241-8668

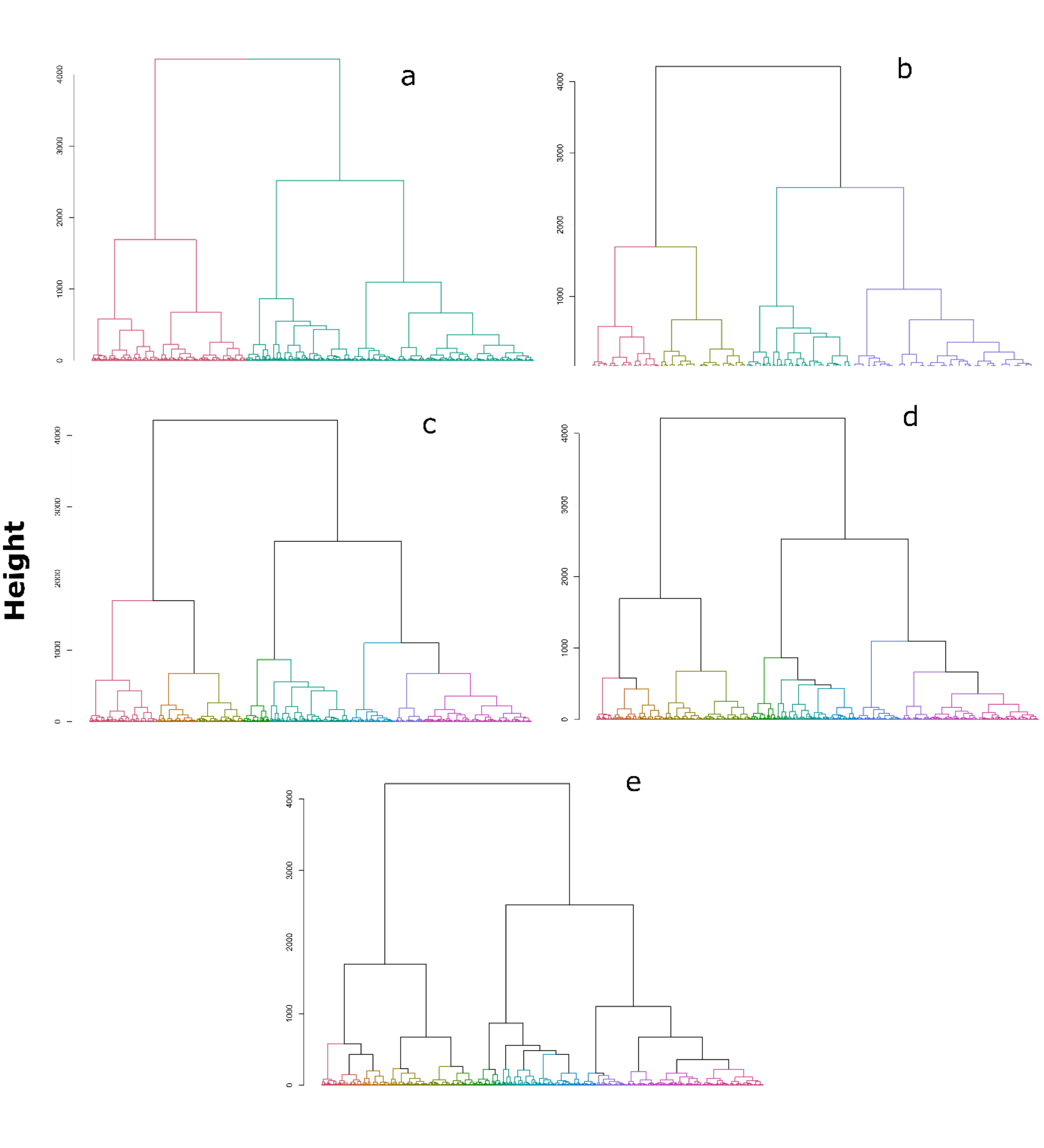

Figure S3-1. Dendrograms of Ward's agglomerative clustering procedure with branches color-coded according to the number of clusters: (a) 2 clusters, (b) 4 clusters, (c) 8 clusters, (d) 14 clusters, and (e) 30 clusters.
